# Beyond the Chair: Exploring the Boundaries of Teledentistry

**DOI:** 10.7759/cureus.62286

**Published:** 2024-06-13

**Authors:** Medhavi Malpe, Sonali G Choudhari, Nikhilesh Nagtode, Pramita Muntode Gharde

**Affiliations:** 1 Department of Community Medicine, Jawaharlal Nehru Medical College, Datta Meghe Institute of Higher Education and Research, Wardha, IND

**Keywords:** dental clinic, teleconsultation, health technology, public health dentistry, telemedicine

## Abstract

Teledentistry, a blend of dentistry and telecommunications used to provide dental care from a distance, holds great promise in enhancing public health, especially in reaching communities with limited access. However, putting it into practice has its own set of obstacles and background variables. It is frequently difficult to obtain dental treatment, especially in rural or economically underdeveloped areas. By removing geographical constraints and offering virtual consultations and diagnostics, teledentistry provides a solution. The quick development of technology, such as portable electronics and high-speed internet, has increased accessibility and efficiency in communication, which has aided in the acceptance of teledentistry. Getting traditional dental care can be costly, particularly for people without insurance. As an affordable substitute for traditional dental care, teledentistry may lower overhead expenses related to physical dental offices. Early interventions and preventive care can greatly enhance oral health results and lessen the need for later, more invasive procedures. There are disparities in the availability of digital gadgets and internet connectivity. The laws that control telehealth services might differ greatly between nations and areas. While teledentistry can help with remote consultations and triage, it might not be able to do thorough examinations or other diagnostic treatments that need in-person attendance. Delivering dependable, top-notch telemedicine services in developing areas can pose challenges. Certain dentists might hesitate to utilize teledentistry due to concerns about the quality of care, liability issues, or potential impacts on their traditional practice models. It could be difficult to get patients to accept and use teledentistry services. This review is undertaken to assess the effectiveness of teledentistry in public health as well as legal and regulatory considerations for practicing teledentistry. Teledentistry might be the future of the oral health sector, thus fully capitalizing on this enormous opportunity to change how oral therapies are administered. Patients, dentists, and the dental community at large will need to have highly open minds. For the community's well-being and to gain its trust, ethical considerations are crucial. The outcomes of teledentistry can be attained by overcoming the obstacles and using comprehensive methods and approaches. It was noted that teledentistry is a potential strategy that combines dental care with telecommunication technology to enhance patient outcomes, reduce healthcare inequalities, and expand access to oral health services. The delivery of oral healthcare is being revolutionized by teledentistry, especially in light of contemporary issues including geographic restrictions, lack of access to dental treatment, and the ongoing global health crisis.

## Introduction and background

Technology advancements in communication and the use of electronic data in remote health services have given rise to the terms "telehealth" and "e-health" [1]. Because computer technology is being used in place of direct, in-person doctor-patient connection, this new approach to managing dentistry is now conceivable [2].

Teledentistry is a specialized field that combines dentistry and digital/telecommunication technologies to address dental health and related difficulties [3]. Since 1994, teledentistry has evolved as a way for dental practitioners to interact virtually. It enables the cooperation of several professionals concerning a patient and the required care for this patient [4]. Before the invention of Information and Communication Technology (ICT), dentists would frequently interact over the phone or transfer clinical data of their patients via telegraph, fax, or mail. Interaction between practitioners and patients through telemedicine has become faster and more efficient because of the internet's accessibility and the ICT’s quick development [5].

A 1994 United States (US) effort to evaluate the oral health of US Army soldiers served as the basis for the first practical implementation of teledentistry [6]. "Oral health is essential to general health and quality of life," said the WHO 2012 cover sheet on oral health. It is the absence of pain in the mouth and face, cancer of the mouth and throat, oral infections and sores, periodontal disease (gum disease), tooth decay, tooth loss, and other illnesses and conditions that hamper a person's ability to bite, eat, smile, talk, and maintain their psychosocial well-being. In developing countries like India, oral infections are common, notably among rural dwellers [7]. So, teledentistry is receiving importance for several reasons. The emerging field of telehealth, which includes teledentistry, is expanding quickly and is already having a significant influence on healthcare [8]. Programs for teledentistry have mostly been used for consultations and expert referrals. There have been prior attempts to diagnose pathological disorders with this technology [9]. Both dental students and practitioners will benefit from the new chances that teledentistry offers to improve conventional teaching techniques in dental education [10]. Dental treatment can be provided by teledentistry in remote and rural locations where access to medical consultations is limited. An ideal setting for the successful use of teledentistry is a growing nation such as India, with its wide-ranging geography, sizable rural population, and well-established healthcare delivery infrastructure enhanced by telecommunications technology [11].

Teledentistry entails the local dentist digitizing and electronically transmitting drawings, diagrams, photographs, and X-rays to a specialist for further analysis or consultation [12]. The prefix "tele" might be added to a variety of healthcare activities to create tele-activities like teleconsultation, tele diagnosing, telecare, tele surveillance, etc. All medical specialties, including teleradiology, telepathology, teledermatology, etc., might simultaneously acquire the same prefix and transform into tele-medical specialties [13]. As it encompasses a variety of technologies and uses, telemedicine is not a single, cohesive discipline [14]. For the most part, to deal with the COVID-19 pandemic, dentists began practicing teledentistry in 2021. This approach is focused on tele-education and teleconsultation. Over the past 10 years, there has been a significant increase in the adoption of tele-technology within the medical and dental sectors, offering a swift, secure, and practical means of disseminating and exchanging health-related data. Teledentistry, in particular, streamlines referral processes by prioritizing consultation time, minimizing waitlists, aiding in decision-making, and addressing patients' requirements more effectively [15]. Even though there are worries about data security and privacy, a noteworthy portion of respondents in a recent survey conducted in Saudi Arabia agreed that teledentistry could strengthen dental practice by building up peer communication, offering guidance, and referring new patients [6].

According to Estai et al., more than 65% of practitioners believe that patients in isolated or rural areas would well-being from a teledentistry system. Additionally, it was noted that respondents expressed willingness to communicate with both their patients and colleagues, with 80-90% indicating that teledentistry would enhance communication with peers and patients. Despite the area of teledentistry’s rapid growth, there are still obstacles preventing its wider application in therapy. Managing costs, data security, and time are the three most significant issues facing teledentistry practices [5].

## Review

Methodology

This review discusses the effectiveness of tele-dentistry and legal and regulatory considerations for practising tele-dentistry. We searched online databases like PubMed (Medline), Google Scholar, and Embase as well as websites and grey literature. The PubMed search strategy was as follows - ("teledentistry"[Title/Abstract] OR "tele-dentistry"[Title/Abstract] OR "telemedicine"[Title/Abstract] OR "tele-medicine"[Title/Abstract] OR "teleconsultation"[Title/Abstract] OR "public health technology"[Title/Abstract] OR "telemedicine"[MeSH Terms]) AND ("Oral Health"[Title/Abstract] OR "Dental care"[Title/Abstract] OR "Dental medicine"[Title/Abstract] OR "dentist*"[Title/Abstract] OR "Dental"[Title/Abstract] OR "dentistry"[MeSH Terms]). The duplicates, abstracts, unpublished works, works written in languages other than English, and articles beyond the scope of the review were excluded.

Discussion

Types

**Figure 1:**
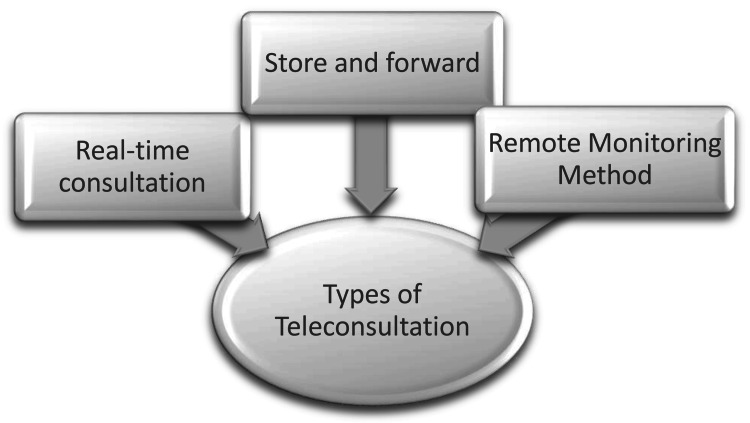
Types of teleconsultation Author credit: Medhavi Malpe

Teleconsultation can be done in three ways. The figure below depicts the type of teleconsultation (Figure 1).

Real-time consultation (synchronous teleconsultation): This involves direct, live interaction between the patient and healthcare provider. This type of teleconsultation replicates the traditional face-to-face visit through the use of technology, enabling immediate communication and response. It transfers the information immediately [16].

Store and forward (asynchronous teleconsultation): This involves transmitting medical data from the patient to the healthcare provider, such as images, videos, laboratory results, and other pertinent information. The provider reviews the information and responds at a later time. This method does not require the simultaneous presence of both parties, making it highly flexible [16].

Remote monitoring method: Patients are regularly checked on while at home or in a hospital. Medical records and other health information are electronically moved from one location to another to provide clinical and administrative support. It enhances healthcare delivery by providing continuous oversight, enabling proactive care, and improving health outcomes [11].

Telecommunication Technologies

**Figure 2:**
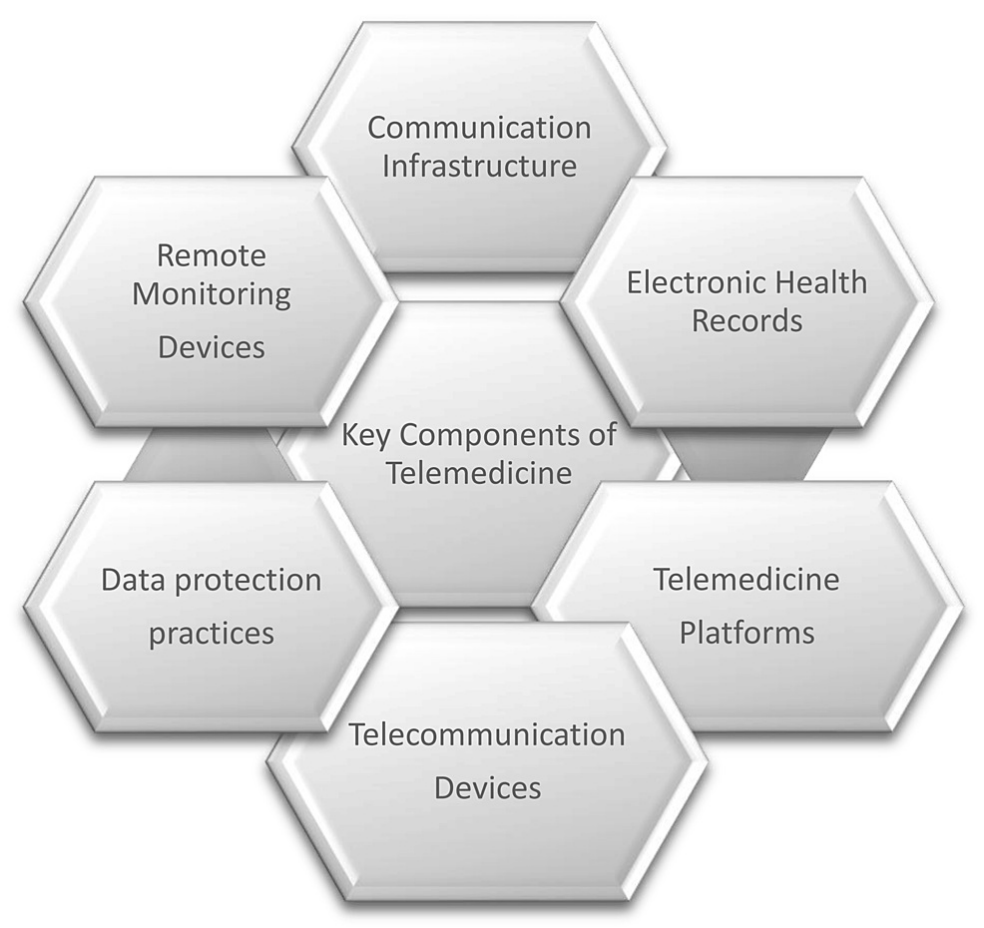
Key components of telemedicine Author credit: Medhavi Malpe

The first difficult query while organizing a telemedicine network is, "What is bandwidth?" The ability to send bits via a communication medium's channels at a speed determined by bandwidth. For a certain degree of system performance, bandwidth is proportionate to data complexity. Integrated Services Digital Network is a dial-up digital connection to the telecommunication carrier, utilized on a call-by-call basis. It can transmit data nearly five times faster than analogue modems over Plain Old Telephone Service. It is the most widely available telecommunication technology worldwide, transmits data at up to 56 kbps and is suitable for audio conferencing, store-and-forward communication, Internet use, and low bandwidth videophone conferencing [17]. According to Raucci-Neto et al., irrespective of dental specialization or prior practice experience, the most often utilized teledentistry technologies were video calls and text messaging. Secure messaging systems ensure private communication, and telephony is used for voice consultations and follow-ups. These technologies collectively enhance access to dental care, improve patient outcomes, and reduce healthcare disparities [18]. The foundation of contemporary telemedicine systems is the Internet, which is quick, efficient, and capable of carrying significant volumes of data. The Internet is the foundation for all modern teledentistry technologies and forms of remote consultation. Modern Internet technology provides online video conferencing, streaming surgeries and treatments, and online training courses for continuing dental education [3]. The figure below shows the components of telemedicine (Figure 2).

The Assistance of Teledentistry

It can offer a less scary, more affordable, and simpler option to get in reach with dentists [16]. Teledentistry can improve oral healthcare delivery, reduce associated expenses, and increase the approach to dental treatment. It also can end the disparities in oral healthcare that exist between urban and rural areas. Patients of primary care physicians who use teleconsultation to access a wide range of skills would benefit greatly from it [3]. According to Chen et al., since it offers an instant response, the interactive videoconferencing approach is more fruitful than the other two primary types of telemedicine in education [10]. A consultation between a dental surgeon, specialized experts, and a patient is possible in real-time method using video calling or conferencing [19].

According to Qari et al., because it was convenient for them to save money on travel, and save, patients favoured virtual visits over regular visits [20]. Teledentistry is most cost-effective in rural places when there is a demand for costly transportation that can be avoided. Patients can get E-prescriptions by using teledentistry. It is very helpful to the elderly, impoverished, and disadvantaged in a place like India. With the extra benefit of less patient concern and a lower risk of hospital-acquired infections, the majority of these patients may skip hospital-based therapy completely and the requirement for costly travel [7]. In addition to enhancing the standard of patient treatment, new information technology (IT) has made it feasible for patients to get full or partial care hundreds of kilometres distant from medical facilities or licensed dentists [3]. By offering peer contact, specialized assistance, and postgraduate study, practitioners' isolation is minimized. Preauthorization, second opinions, and other insurance processes will be completed online very immediately, with the usage of actual dental condition pictures as opposed to textual descriptions and tooth charts [8].

Need for Teleconsultation

Teledentistry is a contemporary approach to dentistry that integrates digital imaging, electronics, health records, and telecommunications technology through an Internet connection. This allows patients to receive care in remote locations and also allows the distant specialist to make an accurate diagnosis, recommend the best course of treatment, and, if necessary, refer patients who are difficult to see [2]. The most popular type of teledentistry is teleconsultation, in which clients or nearby medical professionals use telecommunication to consult with dental specialists. A teleconsultant may be accessed by any doctor or dentist with an efficient desktop computer, one of numerous software applications for teleconsulting, a broadband connection to the Internet, a digital camera, a radiograph/text scanner, and the bare minimum of training. Its application in dental traumatology has been proven to offer patients crucial support in situations where a dentist is not immediately present or accessible. In addition to changing or improving computer-aided design and manufacturing (CAD-CAM) processes, a digital workflow of various strategies has made it possible to fully digitally design complete jaw reconstructions in oral and maxillofacial surgery as well as to fully digitally plan the rehabilitation of masticatory function based on implants [21]. Adding high-resolution digital photos, audio and video clips, and digitalized magnetic resonance imaging (MRI), computed tomography (CT) scan, or standard radiographs may require an extra transmission time, depending on the size and quantity of images to be sent (though this isn't always a problem because most tele-consults aren't happening in real-time) [22]. It has been beneficial in providing consultations for patients with physical and intellectual disabilities, as well as those from prisons and elderly care institutions [12]. Castro Filho discovered statistics showing that telemedicine use cut the frequency of referrals to medical specialists in half [23]. In research on general dentists' and oral and maxillofacial surgeons' opinions on teledentistry, Wood et al. found that most participants thought teledentistry made it easier for patients to get treatment, and they found this accessibility to be provided at a reasonable cost [24].

According to Kern, of the assessment studies that dealt with telemedicine, 76% of them included the evaluation of teleconsultation systems, and 8% of them evaluated health information systems, respectively [13]. It appears that we should keep finding the circumstances and settings under which it is suitable, along with the best channel of communication for each, whether or not they are synchronistic [25].

Ethico-Legal Aspects in Practicing Teledentistry

In addition to its perks, the quickly changing landscape of digital health technology presents social, legal, policy, and financial issues that might prevent it from being extensively implemented. Teledentistry requires resolving several ethical and legal challenges, including data security obtaining informed consent and navigating licensure restrictions. Maintaining the quality of care, resolving access inequities, and adhering to ethical procedures are critical. Accurate paperwork and monitoring insurance coverage are also essential. These methods build trust and safety among patients and practitioners [26]. In past times, practitioners could freely interact and share knowledge with peers in different states. When adopting these techniques, a certain amount of confidentiality was naturally anticipated because the information was shared with just one person or office. As a result, professionals who perform telemedicine or teledentistry need to hold a license in each state. Despite the best efforts of the treating physician or dentist, patients should be told that there is a chance unauthorized individuals may gain access to their medical or dental records. Some of the ethical problems include maintaining security during online transactions and internet fraud. The practitioner has some options for increasing the difficulty of transmission acquisition. Regular, secure data backups are essential for protecting patient records and avoiding negligence suits. If patient data is lost and cannot be recovered due to a lack of backup, the practice owner may be held liable for negligence [27]. The absence of clear standards is the main cause of these issues [3]. To handle the scenario that may develop during teledentistry consultations, dentists must have protocols in place. These protocols should include collaborating with nearby healthcare professionals for in-person care as necessary [21].

Obstacles Inhibiting the Adoption of Teledentistry

With teledentistry, visual inspection, and patient-reported symptoms are frequently used instead of a thorough physical examination of the patient's oral cavity, as in the case of in-person dental visits. For complicated dental diseases in particular, this constraint may have an impact on the precision of diagnosis and treatment planning. Cost of the telecommunication equipment has also been a matter of concern [3]. Variability in patient outcomes and service quality might result from the lack of defined standards and guidelines for teledentistry services [28]. Some dental professionals require specialized training for practising teledentistry. Some dentists and patients do not accept the digital way of treatment because of traditional choices. It turns out to be impracticable to use an online consultation to evaluate extraoral and intraoral swelling, extremely carious teeth, soft tissue lesions, mobile teeth, broken prosthodontic work, and orthodontic problems [19]. Teledentistry faces several obstacles, such as patients' lack of IT literacy, dentists' low IT literacy, patients' unfamiliarity with technology, infrastructure limitations (such as inadequate internet access, hardware shortages, high equipment costs), low video quality, audio-video mismatches that can be resolved through direct communication (simultaneous communication) [29]. Lack of adequate resource investment, lack of system integration, and internet failures were relevant restraining factors. The teleconsultant guidelines contributed to general dentistry’s better decision-making regarding treatment, urgency of case management, and prioritization of referrals to the specialist should be made [30]. The advancements in telemedicine are essential tools for providing patients with more effective and affordable treatment, not a means of dispensing with human jobs [31]. Due to budgetary limitations and a lack of administrative vision, teledentistry has not yet been fully integrated into the mainstream oral healthcare system [32].

Teledentistry in Public Health

Through the Indian Space Research Organization (ISRO), the Department of Information Technology and the Ministry of Communications and Information Technology initiated a pilot telemedicine initiative in 1999. A little over 1,000 telemedicine nodes were set up by government, private, and charitable trust organizations across the nation. A total of 414 nodes were donated by ISRO, of which 384 were situated in outlying medical care facilities connected to 60 super-speciality hospitals. Additionally, 500 operational centres have been developed by the Apollo Telemedicine Networking Foundation, comprising 115 teleophthalmology centres and 164 electronic urban primary health centres. Approximately 150 franchisees are located across the nation. The primary goals were to strengthen the country's healthcare delivery system. The Pan African e-Network Project (2009-2017) was carried out by India for the betterment of global health. This initiative established important connections for the Voice-over-Internet Protocol (VoIP), video conferencing, Internet, telemedicine, and education [33]. As technology advances, telemedicine applications for epidemiological surveillance - such as geographic information systems (GISs) - are progressively rising to new heights [17]. Teledentistry can enable dental professionals to educate the community in remote as well as approachable areas by using video conferencing and online interactive resources. Teledentistry enhances public health by improving access to dental care in underserved and remote communities. It facilitates early detection, diagnosis, and preventive care through remote consultations and education programs. By reducing travel needs and bridging care disparities, teledentistry makes dental services more accessible, affordable, and efficient. Patients can also receive remote follow-up care by using teledentistry.

Future Directions in Teledentistry

Teledentistry has a promising future lies ahead, as technological developments continue to change the way healthcare is delivered. Artificial intelligence (AI), virtual reality (VR), augmented reality (AR), mixed reality (MR), and extended reality (XR) technological developments have completely changed dentistry, bringing in a new era of accuracy, better patient care, and greater instruction. Instead, then taking the place of human labour, these technologies are essential instruments for providing patients with care that is both more effective and affordable. The use of AI, VR, AR, MR, and XR in dentistry is a game-changer, empowering medical practitioners to provide better treatment at a lower cost. According to Joda et al., VR and AR have been applied to many facets of social life, including industrial processing, entertainment, and marketing. Medical fields are no different, particularly those that focus on surgery and use minimally invasive techniques like endoscopic and laparoscopic surgery. It is yet unknown, nevertheless, how widely adopted and routinely used AR/VR technologies are in dentistry [32]. Perhaps the quickest and most economical approach to reducing the health gap between rural and urban areas is through teledentistry. Given the tremendous advancements in information and communication technology, teledentistry has the potential to provide specialized treatment to even the most remote regions worldwide. If the anticipated scarcity of dentists over the coming decade comes to pass, teledentistry will play a significant role in serving not just our urban and suburban populations, but also those in rural regions. Dental treatment will be better integrated into the broader healthcare delivery system through interprofessional communication. Healthcare professionals and IT experts have reassessed teledentistry as an extremely beneficial tool in healthcare due to advancements in data transfer speed and methods over the past decade [3].

The first remotely executed intervention in Italy utilizing a robot linked to a 5G network was documented in a 2020 publication. The surgical procedure used transoral laser microsurgery on a corpse. The anatomy lab, where the surgeon removed a polyp that had been surgically grafted onto the body's voice cords, was 15 kilometres from the corpse. An experimental 5G network enabled two-way data transfer between the robot and human surgeons. Therefore, it becomes sense to believe that teledentistry has just begun to realize just a small part of its full potential and that most dental procedures will soon be able to be completed remotely. Information networks and simulations that enable the remote transmission of skills will provide new tools to the dental profession for the upcoming generation. How digital healthcare develops and advances will depend on everyone's capacity to comprehend and utilize these technologies [2]. Table 1 shows the list of included studies in the review.

**Table 1:** List of included studies in the review

Sr No	Author	Year	Type of article	Finding
1	Raja et al. [1]	2022	Original article	This study shows the key concerns of uncertainty regarding teledentistry’s application across all dental specialties, poor infrastructure, inadequate training, and connectivity problems.
2	Fornaini and Rocca [2]	2022	Review article	Teledentistry may be the oral health industry's future, and to take advantage of this significant chance to completely transform the way oral treatments are carried out, dentists, patients, and the dental community as a whole will need to be very open-minded.
3	Estai et al. [5]	2016	Original article	The findings of this study suggest that the idea of teledentistry and its incorporation into present dental practices were seen with hope and support by dental practitioners in general.
4	Modak and Basu [7]	2020	Review article	This review shows that teledentistry can be an effective tool for managing patients with oral disorders efficiently.
5	Bhambal et al. [8]	2010	Review article	This study shows the applications of teledentistry in various branches of dentistry. The study also concerns the legal issue of practising teledentistry.
6	Singhal et al. [31]	2023	Review article	To fully realize their potential in oral health care, the given article concentrates on utilizing these instruments in pathology, radiography, surgery, and oral medicine. This will ensure a more hopeful and technologically advanced future for dentistry.
7	Maqsood et al. [6]	2021	Research article	According to the provided study, the dental professionals who took part in it exhibited sufficient knowledge and a favourable look toward the use of teledentistry.
8	Kumar et al. [16]	2019	Review article	By giving primary care physicians quick access to effective consultations and by supporting postgraduate and ongoing dental education programs, teledentistry opens up new prospects for health education.
9	Dasgupta and Deb [17]	2008	Review article	This study states that telemedicine is a "forward step in a backward direction," or, in the words of Neil Armstrong, "one small step for introduction technology but one massive leap for Healthcare," but only time will tell.
10	Qari et al. [20]	2024	Original article	Due to its ease of use and ability to save time, money, and effort, patients favoured virtual visits over traditional ones.
11	Jampani et al. [3]	2011	Review article	This study describes that, with all the technical breakthroughs in teledentistry, Dentists could potentially link up with online dental health clinics, leading to a revolutionary era in the field of dentistry.
12	Raucci-Neto et al. [18]	2022	Research article	The results of this study showed that Brazilian dental surgeons were a bit cautious about offering remote dental care support.
13	Bhargava et al. [27]	2020	Review article	This study demonstrates that teledentistry provides affordable distant dental treatment to overcome the scarcity of specialists in rural locations.
14	Mahdavi et al. [29]	2022	Research article	It has made teledentistry possible, enabling live and responsive patient examinations, videoconferencing, patient education from a distance, remote assessment, telemonitoring of the treatment process, and teleconsultations - all of which have ultimately refined patient satisfaction.
15	Kharbanda et al. [33]	2019	Review article	This research demonstrates the need for increased public awareness about the advantages of using telehealth for medical appointments, education, and post-operative follow-up.

## Conclusions

With the advancements in telemedicine and teledentistry, telehealth care may dominate the new era. Teledentistry is a possible approach to improve patient outcomes, decrease healthcare inequities, and increase access to oral health services, particularly in underserved or remote areas, through the combination of telecommunication technology and dental treatment. Teledentistry is leading the way in transforming the provision of oral healthcare, especially in light of contemporary issues including restricted access to dental treatment, geographic constraints, and the ongoing global health crisis. However, addressing ethical considerations is crucial not only for protecting patients but also for fostering trust in teledentistry among both patients and healthcare providers.
